# Academic discipline and university students’ well-being: a comparative analysis with implications for physical and psychological health promotion strategies

**DOI:** 10.3389/fpsyg.2026.1726043

**Published:** 2026-03-17

**Authors:** Zhangyu Yang, Gracia Cristina Villodres, Chen Chen, Xing Zhang, Li Huang, José Joaquín Muros

**Affiliations:** 1 Department of Didactics of Body Expression, Faculty of Educational Sciences, University of Granada, Granada, Spain; 2 College of Physical Education, Fuyang Normal University, Fuyang, Anhui, China; 3 Department of Physical Education and Sport, Faculty of Sport Sciences, University of Granada, Granada, Spain; 4 Institute of Sports Science, College of Physical Education, Southwest University, Chongqing, China

**Keywords:** academic discipline, HRQoL, mental health, physical activity, resilience, self-esteem, university students

## Abstract

**Background:**

The university years represent a formative period for establishing health behaviors and psychological resources. While existing research often examines individual predictors of student well-being, few studies have explored how academic discipline shapes these patterns. The present study aimed to examine differences in physical activity (PA), self-esteem, resilience, health-related quality of life (HRQoL) and negative psychological outcomes (anxiety, stress, and depression) among Chinese undergraduates according to academic discipline.

**Methods:**

A cross-sectional online survey was conducted in December 2024 among 1,560 students from six comprehensive universities in China. Participants were categorized into four academic disciplines: physical education (PE), health sciences (HS), education sciences (ES) and other non-health sciences (ONS). Validated instruments were used to assess PA, self-esteem, resilience, HRQoL and negative psychological symptoms.

**Results:**

Significant differences were observed between academic disciplines. PE students reported the highest scores for PA engagement, self-esteem, resilience and HRQoL, along with the lowest scores for anxiety and stress. In contrast, ES and ONS students reported higher levels of anxiety, stress and depression, as well as lower self-esteem, resilience and HRQoL. Further analyses revealed that male students, those with higher perceived socioeconomic status (PSES) and those with higher PA levels demonstrated more favorable psychological profiles.

**Conclusion:**

Academic discipline, alongside demographic and behavioral factors, plays a key role in shaping students’ psychological health and lifestyle behaviors. University-based health promotion strategies should incorporate academic discipline and prioritize action-based interventions to foster sustainable well-being across diverse student populations.

## 1 Introduction

The university period represents a pivotal stage in the development of individual health literacy and behavioral patterns. The attitudes and lifestyles formed during this phase often exert long-term influences on one’s habits and mental health going into adulthood (Liu et al., 2022). However, with the rapid expansion of higher education in China, college students are now facing mounting physical and mental challenges. According to recent reports, the graduating class of 2025 will comprise approximately 12.22 million students, an increase of 430,000 from the previous year, marking an unprecedented surge in competition within the labor market (Wang and Hu, 2025). This intensifying pressure has been linked to increased academic burden, career-related anxiety and sedentary behavior, and reduced physical activity engagement, along with growing rates of depression, anxiety and academic stress among students (Liu et al., 2022; Zhang et al., 2024). Importantly, these health risks are not solely the result of individual differences given that they may also be shaped by educational contexts. Variations in academic disciplines, curricular intensity and the extent to which health education is integrated into coursework may subtly influence students’ health perceptions and behavioral inclinations, thereby affecting their overall mental health (García-Perez et al., 2023).

Physical activity (PA), as a positive health behavior, has been widely recognized for its significant role in promoting mental health (Herbert, 2022). A substantial body of evidence indicates that regular engagement in PA can effectively reduce anxiety, depression and stress (Liu M. et al., 2024), while simultaneously enhancing self-esteem (Nie et al., 2025) and resilience (Husain et al., 2024). Resilience is conceptualized as an individual’s capacity to maintain psychological stability and adapt effectively in the face of adversity or stressful academic environments. Recent research suggests that self-esteem, defined as a global evaluation of one’s self-worth, is closely linked to mental health outcomes. Moreover, resilience may function as a mediating factor in the relationship between self-esteem and psychological health (Cui et al., 2024) by enabling individuals to cope more effectively with stress and negative emotions such as depression and anxiety (Fan et al., 2024). In addition, HRQoL, which is a multidimensional construct that reflects both physical and mental health status, has been shown to correlate significantly with PA and various psychological resources (Liu R. et al., 2024). These interconnections suggest that enhancing PA engagement among university students may, not only, contribute to better physical health, but, also, serve as a pathway to improved psychological resources and, ultimately, higher HRQoL (Cui and Zhang, 2022).

Growing attention has been paid to the way in which structural differences between academic disciplines (such as curricular demands, assessment formats and educational values) may bring about systematic variations in students’ physical activity engagement, psychological resources and emotional well-being. Extensive research indicates that health science students, including Medicine and Nursing students, are often considered a high-risk group for mental health issues given that they exhibit high levels of anxiety and depression (Brambila-Tapia et al., 2020; López-García et al., 2018). However, previous research has also identified substantial psychological distress among students enrolled on non-health science subjects, including Engineering, Law, and the Humanities and Social Sciences, with some reporting levels of anxiety and depression that are comparable to those exhibited by Health Sciences students (Lipson et al., 2016). Moreover, academic discipline may influence the way in which students conceptualize PA, with some deeming it to be a utilitarian behavior and others an academic endeavor. This shapes student engagement patterns and emotional regulation capacities (Nowak et al., 2019). Evidence suggests that students with greater health knowledge and consistent exercise habits are more likely to adopt positive health behaviors and attitudes (Blandón et al., 2017; García-Perez et al., 2023). In consideration of all of that presented above, it is suggested that whilst academic disciplines differ structurally, they may also be systematically differentiated in terms of health-related cognition, behavioral tendencies and psychological risk. This provides both theoretical and practical justification for exploring the relationships between PA engagement, self-esteem, resilience, HRQoL and mental health through the lens of academic discipline.

Although numerous studies have explored mental health and behavioral patterns across academic fields, evidence remains equivocal and a cohesive understanding is still lacking. In order to address this gap, the present study aimed to systematically compare students from four academic disciplines [physical education (PE), health sciences (HS), education sciences (ES), and other non-health sciences (ONS)] in terms of key psychological and behavioral health indicators, including PA engagement, self-esteem, resilience, anxiety, stress, depression and HRQoL. Through consideration of academic discipline as a central contextual variable, the present study will produce data on the influence of variations in curricular structure, health knowledge exposure and educational focus. This may provide empirical evidence regarding academic discipline disparities and serve as a foundational basis to recommend targeted mental health promotion and student support strategies in diverse university settings.

## 2 Materials and methods

### 2.1 Participants and procedures

A cross-sectional survey was conducted in December 2024 at six comprehensive universities located in China. The target population comprised full-time undergraduate students. Participants were categorized into four academic disciplines based on the degree on which they were enrolled: Physical Education (PE) (15.9%), Health Sciences (HS) (9.2%), Education Sciences (ES) (35.6%) and Other Non-Health Sciences (ONS) (39.3%). This classification approach was adapted from previously peer-reviewed literature (García-Perez et al., 2023), as well as by the structure of university curricula and the extent to which health-related knowledge and PA exposure are embedded within each academic field. Specifically, the PE group included majors such as physical education, sport training, and exercise science, which involve substantial practical training and regular physical activity. The HS group comprised disciplines including medicine, nursing, public health, and rehabilitation sciences, characterized by formal health knowledge but limited structured physical training. The ES group included programs such as pedagogy, psychology, and educational management, which focus primarily on theoretical and psychosocial training. Finally, the ONS group encompassed majors unrelated to health or education, including engineering, law, computer science, and business administration.

Prior to data collection, the research team conducted an extensive literature review and selected a set of psychometrically validated instruments that were appropriate for gathering data on the variables of interest. The final questionnaire was digitalized using the Sojump platform^1^, and a digital poster featuring a brief study description and a QR code was created to facilitate distribution. For participant recruitment, the research team collaborated with 21 university instructors. The questionnaire was disseminated via a dualchannel strategy, which combined educational platforms and social networking tools such as WeChat and QQ, as well as inperson promotion during lectures. All participants were required to complete an informed consent form prior to beginning the survey, which emphasized voluntary participation, data anonymity and the right to withdraw at any time. No financial or material incentives were provided to respondents. Given that the survey was accessible online, several measures were implemented to ensure data quality and to minimize duplicate or invalid responses. The platform restricted multiple submissions from the same device and IP address. In addition, four attention-check items were embedded in the questionnaire to identify inattentive responses or careless responding. To ensure ethical compliance, the study adhered to the principles outlined in the Declaration of Helsinki and was approved by the Ethics Review Committee of Southwest University (SWUETH20230912003).

A total of 3,200 questionnaires were initially collected. During data screening, responses were excluded if they met any of the following criteria: (1) excessively short completion times; (2) substantial missing data; and (3) failure to pass logic consistency checks or any of the attention-check items. After applying these quality control procedures, 1,640 questionnaires were removed. Ultimately, 1,560 valid responses were retained for analysis, including 434 males (27.8%) and 1,126 females (72.2%).

### 2.2 Instruments and measurements

#### 2.2.1 IPAQ-SF

Physical activity was assessed using the International Physical Activity Questionnaire–Short Form, a widely used self-report instrument consisting of seven items. The scale captures the frequency and duration of PA engaged in over the 7 days prior to questionnaire completion according to three intensity levels (vigorous, moderate and walking) (Craig et al., 2003). Weekly PA was calculated by multiplying MET values by the number of days and minutes engaged in each activity. Total PA was expressed as the sum of all MET-minutes per week and categorized as low, moderate, or high intensity based on standard scoring criteria. The instrument also recorded average daily sitting time and has demonstrated acceptable validity among Chinese university students (Macfarlane et al., 2007).

#### 2.2.2 Depression, Anxiety and Stress Scale

Negative emotional states were assessed using the Depression, Anxiety and Stress Scale–21 Items (DASS-21). This self-report instrument was conceived to measure symptoms across three domains: depression, anxiety and stress (Lovibond, 1995). The scale comprises 21 items, with each subscale containing seven items. Subscale scores were computed by summing responses to the respective seven items, yielding a total domain score that ranged from 0 to 21 (Gomez, 2016). The DASS-21 has been widely applied in studies evaluating psychological distress in university populations and has been validated for use among Chinese university students (Gong et al., 2010).

#### 2.2.3 Rosenberg Self-Esteem Scale

Self-esteem was assessed using the Rosenberg Self-Esteem Scale. This is a 10-item instrument that comprises five positively framed and five negatively framed items (Rosenberg et al., 1995). Responses are rated along a four-point Likert scale, with reverse scoring being applied to negatively phrased items. Total scores range from 10 to 40, with higher scores reflecting greater self-esteem. The instrument has been widely applied and validated for use among Chinese university students (Leung and Wong, 2008).

#### 2.2.4 Resilience

Resilience was measured using the Connor-Davidson Resilience Scale. This scale comprises 25 items that are rated along a five-point Likert scale ranging from 0 (Not at all) to 4 (Most of the time) (Connor and Davidson, 2003). Total scores are calculated by summing all item responses, with higher scores reflecting greater resilience. The instrument has demonstrated robust psychometric properties and is widely validated for use among Chinese university students (Yu and Zhang, 2007).

#### 2.2.5 Health-related quality of life (HRQoL)

Health-related quality of life (HRQoL) was assessed using the 36-Item Short Form Health Survey. This is a widely validated instrument that comprises two composite scores [Physical Component Summary (PCS) and Mental Component Summary (MCS)] as a means of representing overall physical and mental health. Each subscale score is calculated by averaging item responses within the corresponding domain, resulting in scores that range from 0 to 100. Higher values indicate better perceived health status (Ware, 2003). This instrument has been extensively applied in Chinese university student populations (Lam et al., 2005).

#### 2.2.6 Perceived socioeconomic status

Perceived socioeconomic status (PSES) was assessed using a single self-report item asking participants to evaluate their family’s economic condition compared with their peers. Responses were rated on a 5-point scale ranging from 1 (“very poor”) to 5 (“very good”).

### 2.3 Statistical analysis

Data were analyzed using the statistical software IBM SPSS version 26.0 for Windows. Descriptive statistics (means, standard deviation and percentages) were calculated for all variables. Due to the large sample size, parametric tests were used as, according to the Central Limit Theorem, assumption violations tend to be less relevant when working with large samples (Field, 2018). Student *t*-tests for two group comparisons and analysis of variance (ANOVA) with Tukey *post-hoc* test was employed to analyze differences according to academic discipline. Pearson correlations were performed to examine the strength and direction of the linear relationships between the continuous variables. Significance was set at 0.05.

## 3 Results

Table 1 summarizes psychological outcomes according to sex, perceived socioeconomic status (PSES) and physical activity engagement (PA). Significant differences were observed between males and females. Female students reported higher stress (*p* = 0.005), while male students exhibited higher levels of resilience, self-esteem and HRQoL in both PH and MH domains (*p* < 0.01). No significant sex differences were found for anxiety or depression (*p* > 0.05).

**TABLE 1:** Sample characteristics according to sex, perceived socioeconomic status and physical activity engagement.

Characteristics				%	Anxiety	Comp.	*p*	Stress	Comp.	*p*	Depress.	Comp.	*p*		
Total (*n* = 1560)				100%	3.89 ± 3.16			4.97 ± 3.64			3.18 ± 3.29				
Sex			Men (*n* = 434)	27.8%	3.74 ± 3.40		0.280	4.55 ± 3.67		0.005	3.14 ± 3.47		0.788		
			Women (*n* = 1126)	72.2%	3.94 ± 3.06			5.13 ± 3.62			3.19 ± 3.22				
PSES			Low (*n* = 370)	23.7%	4.58 ± 3.88	Medium	<0.001	5.79 ± 4.16	Medium	<0.001	4.07 ± 4.10	Medium	<0.001		
						High	0.090		High	0.096		High	0.008		
			Medium (*n* = 1122)	71.9%	3.67 ± 2.81	High	0.995	4.71 ± 3.39	High	0.971	2.91 ± 2.92	High	0.957		
			High (*n* = 68)	4.4%	3.71 ± 3.75			4.81 ± 3.96			2.79 ± 3.42				
PA (MET – min/week)			Low (*n* = 518)	33.2%	3.99 ± 3.00	Medium	0.988	5.16 ± 3.57	Medium	0.985	3.57 ± 3.25	Medium	0.097		
						High	0.177		High	0.043		High	0.001		
			Medium (*n* = 512)	32.8%	4.02 ± 3.14	High	0.133	5.12 ± 3.62	High	0.066	3.14 ± 3.22	High	0.283		
						High (*n* = 530)	34%	3.65 ± 3.32			4.62 ± 3.71			2.83 ± 3.37				
**Characteristics**		**%**	**Resilience**	**Comp.**	***p***	**SE**	**Comp.**	***p***		**HRQoL**					
									**PH**		**Comp.**	***p***	**MH**	**Comp.**	***p***
Total (*n* = 1560)		100%	62.61 ± 15.24			30.43 ± 4.78			313.64 ± 58.63				283.68 ± 64.77		
Sex	Men (*n* = 434)	27.8%	66.21 ± 16.53		<0.001	31.05 ± 4.71		<0.001	325.42 ± 58.38			<0.001	299.06 ± 60.36		<0.001
	Women (*n* = 1126)	72.2%	61.22 ± 14.48			30.18 ± 4.79			309.10 ± 58.11				277.75 ± 65.46		
PSES	Low (*n* = 370)	23.7%	59.46 ± 16.31	Medium	<0.001	29.11 ± 5.09	Medium	<0.001	303.07 ± 62.56		Medium	<0.001	269.13 ± 69.85	Medium	<0.001
				High	<0.001		High	<0.001		High	0.019			High	0.006
	Medium (*n* = 1122)	71.9%	63.15 ± 14.68	High	<0.001	30.69 ± 4.59	High	<0.001	316.51 ± 56.99		High	0.574	287.78 ± 62.41	High	0.626
High (*n* = 68)	4.4%	70.75 ± 14.32			33.24 ± 4.18			323.82 ± 56.76				295.19 ± 63.27		
PA (MET – min/week)	Low (*n* = 518)	33.2%	59.00 ± 14.30	Medium	0.002	29.44 ± 4.76	Medium	<0.001	305.82 ± 61.22		Medium	0.048	276.06 ± 65.59	Medium	0.587
				High	<0.001		High	<0.001			High	<0.001		High	<0.001
	Medium (*n* = 512)	32.8%	62.21 ± 14.57	High	<0.001	30.58 ± 4.73	High	0.067	314.40 ± 55.59		High	0.206	280.00 ± 66.11	High	0.001
High (*n* = 530)	34%	66.52 ± 15.86			31.23 ± 4.68			320.54 ± 58.07				294.67 ± 61.20		

PA, physical activity; Depress, depression; PSES, perceived socioeconomic status; SE, self-esteem; Res, resilience; HRQoL, health-related quality of life; PH, physical health; MH, mental health; PSES, perceived socioeconomic status; Comp., comparison.

Perceived socioeconomic status was significantly associated with multiple psychological indicators. Students with lower PSES reported significantly higher levels of anxiety, stress and depression compared to those with medium PSES (*p* < 0.001), whilst outcomes pertaining to the high PSES group were significant only for depression (*p* = 0.008). In contrast, resilience and self-esteem scores were progressively higher in accordance with increasing PSES, with significant differences emerging across all groups (*p* < 0.001). HRQoL scores for both PH and MH domains were also significantly lower in the low PSES group compared to medium and high groups, though no significant differences were observed between medium and high PSES.

Similarly, students with higher PA engagement reported significantly lower levels of stress (*p* = 0.043) and depression (*p* = 0.001) compared to those with low PA. In contrast, no significant differences in anxiety were observed across PA levels (*p* > 0.05). Resilience scores increased significantly across all three PA grouping (*p* < 0.01) with increasing intensity bringing about a concomitant increase in resilience. Self-esteem also varied significantly across PA groups, with students in the low PA group reporting lower scores than those in the medium and high PA groups (*p* < 0.001); however, no significant difference was found between medium and high PA groups (*p* = 0.067). With regards to HRQoL, students with low PA engagement reported significantly lower PH scores than those with medium (*p* = 0.048) and high PA (*p* < 0.001). For MH, significant differences were observed between low and high PA (*p* < 0.001), as well as between medium and high PA (*p* = 0.001), while low and medium groups did not significantly differ (*p* = 0.587).

Table 2 presents the correlation coefficients produced between PA, anxiety, stress, depression, resilience, self-esteem and HRQoL. PA (MET-min/week) was negatively correlated with anxiety (*r* = −0.056, *p* = 0.028), stress (*r* = −0.077, *p* = 0.002) and depression (*r* = −0.089, *p* < 0.001), and positively correlated with resilience (*r* = 0.224, *p* < 0.001), self-esteem (*r* = 0.154, *p* < 0.001), physical health (*r* = 0.099, *p* < 0.001) and mental health (*r* = 0.128, *p* < 0.001). With regards to anxiety, this was positively associated with stress (*r* = 0.782, *p* < 0.001) and depression (*r* = 0.753, *p* < 0.001), and negatively associated with resilience (*r* = −0.345, *p* < 0.001), self-esteem (*r* = −0.426, *p* < 0.001), physical health (*r* = −0.382, *p* < 0.001) and mental health (*r* = −0.485, *p* < 0.001). At the same time, stress was positively associated with depression (*r* = 0.750, *p* < 0.001), and negatively associated with resilience (*r* = −0.357, *p* < 0.001), self-esteem (*r* = −0.451, *p* < 0.001), physical health (*r* = −0.347, *p* < 0.001) and mental health (*r* = −0.554, *p* < 0.001). Also, depression was negatively associated with resilience (*r* = −0.434, *p* < 0.001), self-esteem (*r* = −0.552, *p* < 0.001), physical health (*r* = −0.326, *p* < 0.001) and mental health (*r* = −0.522, *p* < 0.001). In a similar sense, resilience was positively correlated with self-esteem (*r* = 0.660, *p* < 0.001), physical health (*r* = 0.308, *p* < 0.001) and mental health (*r* = 0.451, *p* < 0.001), whilst self-esteem was positively associated with physical (*r* = 0.290, *p* < 0.001) and mental health (*r* = 0.489, *p* < 0.001). Finally, physical health was positively correlated with mental health (*r* = 0.608, *p* < 0.001).

**TABLE 2:** Correlation coefficients between study variables.

	Anxiety	Stress	Depression	Resilience	Self-esteem	HRQoL	
						PH	MH
PA	−0.056*	−0.077**	−0.089**	0.224**	0.154**	0.099**	0.128**
Anxiety	.	0.782**	0.753**	−0.345**	−0.426**	−0.382**	−0.485**
Stress			0.750**	−0.357**	−0.451**	−0.347**	−0.554**
Depression				−0.434**	−0.552**	−0.326**	−0.522**
Resilience					0.660**	0.308**	0.451**
Self-esteem						0.290**	0.489**
HRQoL PH						.	0.608**

PA, physical activity; HRQoL, health-related quality of life; PH, physical health; MH, mental health.

*p < 0.05,

**p < 0.01.

Table 3 presents participant characteristics according to academic discipline. In terms of PA engagement (MET-min/week), PE undergraduates reported being more active than ES, HS and ONS students (*p* < 0.01). In the case of anxiety, ES students reported higher levels than PE (*p* = 0.002), HS (*p* = 0.044) and ONS (*p* = 0.031) students. At the same time, ES students reported higher stress than PE (*p* = 0.001) and HS (*p* = 0.003) students. With regards to resilience, PE undergraduates reported higher resilience than ES (*p* < 0.001), HS (*p* = 0.005) and ONS (*p* < 0.001) undergraduates. Also, PE under-graduates reported higher self-esteem than ES (*p* = 0.003) and ONS (*p* = 0.007) under-graduates. Turning attention to physical health, ES undergraduates exhibited poorer perceptions than PE (*p* < 0.001) and ONS (*p* < 0.001) students. Finally, ES students also reported lower mental health than PE (*p* < 0.001) and ONS (*p* = 0.028) students. However, PE students reported more positive mental health than ONS (*p* = 0.026).

**TABLE 3:** Sample characteristics according to academic discipline.

Characteristics		*N*	%	PA (MET-min/week)	*p*	Anxiety	*p*	Stress	*p*	Depress.	*p*	Resilience	*p*	SE	*p*	HQRoL			
																PH	*p*	MH	*p*
Total		1560	100	3051.21 ± 2378.854	<0.001^1^	3.89 ± 3.16	0.001^2^	4.97 ± 3.64	<0.001^3^	3.18 ± 3.29	0.060	62.61 ± 15.24	<0.001^4^	30.43 ± 4.78	0.004^5^	313.64 ± 58.63	<0.001^6^	283.68 ± 64.77	<0.001^7^
Academic area	PE	248	15.9	6055.44 ± 2765.82		3.44 ± 3.76		4.33 ± 4.01		2.76 ± 3.86		67.43 ± 16.03		31.42 ± 4.60		324.01 ± 58.34		298.60 ± 56.70	
	ES	556	35.6	2488.02 ± 1844.80		4.29 ± 3.32		5.41 ± 3.78		3.33 ± 3.32		61.48 ± 15.56		30.14 ± 5.10		304.52 ± 60.76		274.51 ± 68.11	
	HS	143	9.2	2436.58 ± 1598.09		3.52 ± 2.49		4.24 ± 3.04		2.84 ± 2.53		62.12 ± 14.12		30.53 ± 4.40		310.73 ± 59.30		287.80 ± 63.50	
	ONS	613	39.3	2490.00 ± 1816.58		3.79 ± 2.83		4.99 ± 3.43		3.29 ± 3.16		61.79 ± 14.52		30.26 ± 4.58		318.39 ± 55.44		284.99 ± 63.84	

PA, physical activity; HRQoL, health-related quality of life; PH, physical health; MH, mental health; PE, physical education; ES, education sciences; HS, health sciences; ONS, other non-health science. Statistically significant differences between groups:

^1^ab-ac-ad;

^2^ba-bc-bd;

^3^ba-bc;

^4^ab-ac-ad;

^5^ab-ad;

^6^ba-bd;

^7^ab-ad-bd.

## 4 Discussion

The present study systematically compared university students across academic disciplines in terms of physical activity (PA) engagement, self-esteem, resilience, health-related quality of life (HRQoL) and negative psychological outcomes including depression, anxiety and stress. Findings revealed significant disparities in health behaviors and psychological indicators as a function of academic discipline (PE, HS, ES and ONS). These outcomes suggest that academic background may serve as a meaningful contextual factor when it comes to influencing students’ physical and mental health.

The present study found that PE students exhibited significantly higher levels of PA compared to their peers in other academic disciplines. This finding aligns closely with previous research that uncovered that Spanish university students enrolled on courses linked with PA and sport sciences displayed stronger exercise motivations and higher participation frequency than those in other fields (García-Perez et al., 2023). Similar trends have been confirmed in multiple European studies. For instance, cross-national research involving universities in Poland and Romania consistently demonstrates that PE students report substantially higher levels of PA engagement than their counterparts in other academic disciplines, which reinforces the notion that academic background can play a critical role in shaping students’ PA engagement (Fagaras et al., 2015; Kosiba et al., 2019). This distinction may be partially attributed to the practical nature of the PE curriculum, which emphasizes sustained participation in exercise training, skill development and sport-based instruction. As a result, PE students tend to develop a more favorable understanding of exercise, greater self-efficacy and stronger intrinsic motivation. All of these factors collectively facilitate the formation of regular PA habits (Leyton-Roman et al., 2020). In contrast, although HS students typically receive structured instruction in medical and health-related content, their PA engagement in the present study was comparable to that of their ES and ONS counterparts, with no significant difference emerging between these groups. Prior research suggests that even students with substantial health knowledge do not necessarily engage in more consistent PA than their peers (Sahasakul et al., 2023). This highlights that knowledge does not always automatically translate into healthy behavior (Saintila et al., 2024). These findings underscore the need for universities to move beyond knowledge-based instruction and develop action-oriented strategies for health promotion, particularly for students who are well-informed but demonstrate low behavioral adherence. Such strategies should integrate experiential learning, enhance motivation and foster supportive environments that are capable of transforming health awareness into sustained behavioral practice.

Education sciences and ONS students reported consistently higher levels of stress, anxiety and depression compared to their peers undertaking PE and HS. This finding is in line with previous research and suggests that students in the fields of humanities and education may be more vulnerable to emotional difficulties, possibly due to the structure of their academic programs, heightened interpersonal sensitivity and greater attentiveness to emotional experiences (Brenneisen Mayer et al., 2016; Deb et al., 2016). In addition, the concept of “engineering stress culture” offers further insight into the elevated negative emotional levels observed among students in non-health disciplines, particularly those in competitive fields such as Engineering and Law. This framework suggests that students in such disciplines often perceive intense academic pressure as normative and may downplay the need for psychological support or emotional regulation, which leads to the accumulation of negative affect in the absence of timely intervention (Jensen and Cross, 2019).

Beyond their relatively better performance on negative emotional indicators, the present study reveals PE students also exhibit significantly higher self-esteem, resilience and HRQoL – both in PH and MH – when compared with ES, HS and ONS students. This finding suggests that PE students possess comparatively stronger psychological adaptability and more favorable perceptions of their overall mental health. Such outcomes may be partly attributed to the high levels of self-efficacy and intrinsic motivation commonly observed in PE students, which likely contribute to enhanced self-worth and greater resilience when facing challenges (Leyton-Roman et al., 2020). In contrast, ES and ONS students exhibited lower levels of resilience and HRQoL, which may reflect a combination of higher academic stress, reduced opportunities for PA engagement and weaker internalization of health-promoting behaviors (Jensen and Cross, 2019; Ruiz-Hernández et al., 2022). Resilience, in particular, is widely recognized as a key protective factor associated with reduced levels of anxiety and depression, while also playing a crucial role in buffering academic stress and enhancing life satisfaction (Fang et al., 2025). Further analyses in the present study revealed a consistent pattern across dimensions of HRQoL. Specifically, PE students scored highest on both PH and MH subscales, whereas ES students reported the lowest scores. This implies that higher PA engagement may contribute, not only, to improved physical health perceptions, but, also, to more positive psychological states and emotional experiences (de Santana et al., 2023). Taken together, this finding indicates that academic discipline, aside from its influence on students’ responses to psychological stressors, may also shape their pathways of psychological resource development, thereby indirectly contributing to their overall health.

Further, the present study revealed that PA engagement is positively associated with self-esteem, resilience and both dimensions of health-related quality of life (HRQoL), namely PH and MH, whilst also being negatively related with anxiety, stress and depression. These findings are consistent with prior research and reinforce the role of PA as a positive health behavior that, not only, enhances psychological resources, but, also, helps regulate emotions and buffer the effects of stress (Nie et al., 2025). The psychological benefits of exercise can be explained through both self-efficacy and physiological mechanism frameworks. According to the self-efficacy hypothesis, regular PA engagement is a challenging yet mastery-oriented process. As individuals acquire new skills and achieve exercise goals, they develop a stronger belief in their own abilities, which, in turn, contributes to improved self-esteem and resilience (Gallese et al., 2004). From a physiological perspective, exercise is believed to stimulate the release of neurochemicals such as endorphins, increase cerebral oxygenation, and enhance cardiovascular function, thereby alleviating symptoms of anxiety and depression and promoting overall health (Mohammadi-Nezhad, 2011). Moreover, the present study found significant associations between self-esteem, resilience, HRQoL and negative emotional indicators, which suggests that psychological resources and subjective emotional well-being are deeply intertwined among university students (Smith, 2024).

In addition to academic discipline, the present study further indicated that male students scored significantly higher than their female counterparts in self-esteem, resilience and both dimensions of HRQoL (PH and MH), while reporting lower levels of stress. These findings are consistent with prior research suggesting that female students are generally more susceptible to negative emotional states and tend to report higher stress sensitivity (Liu and Tian, 2024). Furthermore, students with higher PSES demonstrated more favorable outcomes in terms of psychological resources, such as self-esteem and resilience, as well as lower levels of depression. This finding echoes previous evidence linking socioeconomic advantage to better mental health (Guo and Li, 2025), suggesting that demographic and economic backgrounds are critical determinants of psychological vulnerability and resource availability within the university environment.

Present findings deepen our understanding of the multifaceted factors influencing university students’ mental health and offer practical implications for the design of targeted health promotion strategies in higher education. Interventions should move beyond academic discipline distinctions to incorporate gender, socioeconomic status and PA engagement as integral components in the development of more personalized and structured support systems. Universities are encouraged to adopt multi-level strategies to help students internalize health awareness as part of their daily lifestyle. Such strategies could include enhancing motivational mechanisms, improving access to PA resources and integrating experiential health curricula. Ultimately, such approaches may contribute to long-term improvements in psychological well-being and quality of life.

### 4.1 Limitations and future directions

Although the present study provides initial evidence on existing associations between physical activity, self-esteem, resilience, HRQoL and negative psychological outcomes across different academic disciplines, several limitations should be acknowledged. First, due to the cross-sectional design employed; causal relationships cannot be established. Thus, future longitudinal or experimental studies are needed to confirm the directionality of observed associations. Second, all data were collected using self-reported measures, which may be subject to recall bias and social desirability effects. The use of objective assessments, such as wearable device tracking of physical activity engagement, would enhance measurement accuracy. Third, the sample was drawn from six universities in Anhui province, China, which may limit the generalizability of findings. Future studies should include more diverse and geographically representative samples to enhance external validity.

## 5 Conclusion

The present study underscores the significant impact of academic discipline on university students’ PA engagement, self-esteem, resilience, HRQoL and negative psychological outcomes. Specifically, students in PE reported the highest levels of physical activity, resilience, and self-esteem, whereas those in ES and ONS exhibited the highest levels of anxiety, stress, and depression. These quantitative findings provide an essential empirical foundation, highlighting the importance of considering academic discipline when designing future mental health and PA interventions within university settings. Educational institutions should move beyond knowledge-based instruction and promote action-oriented programs that foster lasting behavior change. Tailored approaches that account for academic discipline, PA, gender and socioeconomic diversity are essential to enhance the psychological wellbeing and health outcomes of students.

## Data Availability

The raw data supporting the conclusions of this article will be made available by the authors, without undue reservation.

## Footnotes

1
www.sojump.com
